# A case of bilateral revision total knee arthroplasty using distal femoral allograft–prosthesis composite and femoral head allografting at the tibial site with a varus-valgus constrained prosthesis: ten-year follow up

**DOI:** 10.1186/s12891-018-1981-2

**Published:** 2018-03-02

**Authors:** Sung-Hyun Lee, Sung-Hyun Noh, Keun-Churl Chun, Joung-Kyue Han, Churl-Hong Chun

**Affiliations:** 1 Department of Orthopedic Surgery, School of Medicine, Wonkwang University Hospital, Muwang-ro 895, Iksan, Jeollabuk-do South Korea; 2 College of Sports Science, Chung-Ang University, Anseong, South Korea

**Keywords:** APC, Structural allograft, RTKA, Bilateral knee

## Abstract

**Background:**

We report the successful use of allograft–prosthesis composite (APC) and structural femoral head allografting in the bilateral reconstruction of large femoral and tibial uncontained defects during revision total knee arthroplasty (RTKA).

**Case presentation:**

A 67-year-old female with degenerative arthritis underwent bilateral total knee arthroplasty (TKA) using the Press Fit Condylar (PFC) modular knee system at our clinic in March, 1996. At 8 years postoperatively, the patient presented with painful, bilateral varus knees, with swelling, limited passive range of motion (ROM), and severe instability. We treated to reconstruct both knee using a femoral head allograft at the tibial site, a structural distal femoral allograft at the femoral site, and a varus-valgus constrained (VVC) prosthesis with cement. At the 10-year follow up, we found no infection, graft failure, loosening of implants, in spite of using massive bilateral structural femoral head allografts in RTKA.

**Conclusion:**

The use of APC enabled a stable and durable reconstruction in this uncommon presentation with large femoral bone deficiencies encountered during a RTKA.

**Electronic supplementary material:**

The online version of this article (10.1186/s12891-018-1981-2) contains supplementary material, which is available to authorized users.

## Background

Bone loss in revision total knee arthroplasty (RTKA) presents a significant surgical challenge because of the need to maintain proper alignment while establishing a stable bone-implant interface. The management of femoral and tibial bone loss is crucial for a successful RTKA, and the severity and location of bony defects determine the optimal type of reconstruction. Options for reconstruction of large defects include metal augments, custom prostheses, massive autogenous bone-grafts and massive allografts [1].

Allografts provide a biological solution, with the advantage of easy fashioning to fit irregular defects, and restore bone stock at a relatively low cost. Allograft–prosthesis composite (APC) are useful for implants when extensive bone loss is present, and are recommended for tibial plateau or femoral metaphyseal deficiency, according to Engh and Parks [2]. The majority of knee surgeons prefer metal augmentation, rotational hinge, and megaprosthesis when extensive bone loss occurs after TKA, but APC is used in this study.

We report a successful case of consecutive bilateral RTKAs using APC for the distal femur and structural femoral head allografting for the proximal tibia in the reconstruction of large femoral and tibial uncontained defects.

## Case presentation

**Fig. 1:**
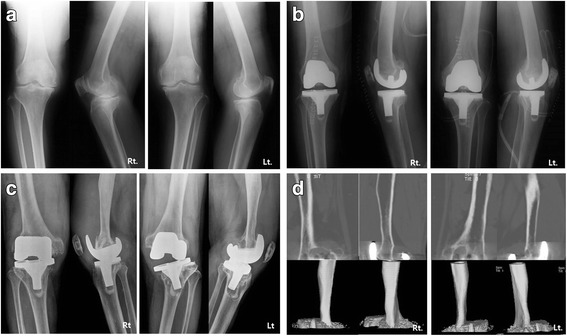
**a** Pre- and **b** postoperative radiographs of primary bilateral TKA; **c** 8-year postoperative radiographs showing extensive osteolysis on both femoral and tibial sides; **d** computed tomography of both knees showing smooth bony erosion on the anterior aspect of the both distal femur

A 67-year-old female with degenerative arthritis underwent bilateral total knee arthroplasty (TKA) using the Press Fit Condylar (PFC) modular knee system (PFC™, Johnson & Johnson Professional Inc., Raynham, MA, USA) at our clinic in March, 1996 (Fig. 1a, b). Normal rehabilitation processes were followed after surgery and the patient was able to walk with normal gait and without complications.

At 8 years postoperatively, the patient presented with painful, bilateral varus knees, with swelling, limited passive range of motion (ROM) (right knee: 0–45°, left knee: 0–90°), and severe instability. The patient was unable to walk and presented in a wheelchair. Anteroposterior and lateral radiographs revealed severe osteolytic bone defects in both the femoral and tibial aspects, along with primary total knee prosthesis and dissociation and subluxation of bilateral implants (Fig. 1c). Moreover, there were severe osteolytic lesions around the femoral prosthesis and along the femoral shaft on computed tomography (Fig. 1d). Preoperative bone scan and laboratory data rulled out infection. We decided to reconstruct the left knee first, using a femoral head allograft at the tibial site, a structural distal femoral allograft at the femoral site, and a varus-valgus constrained (VVC) prosthesis (NexGen LCCK, Zimmer, Warsaw, IN, USA) with cement. Determining the size of the APC to use prior to surgery is a very important and was measured using templating on the previous radiographs. The size of the structural distal femoral allograft was determined using a templating technique before surgery.

**Fig. 2:**
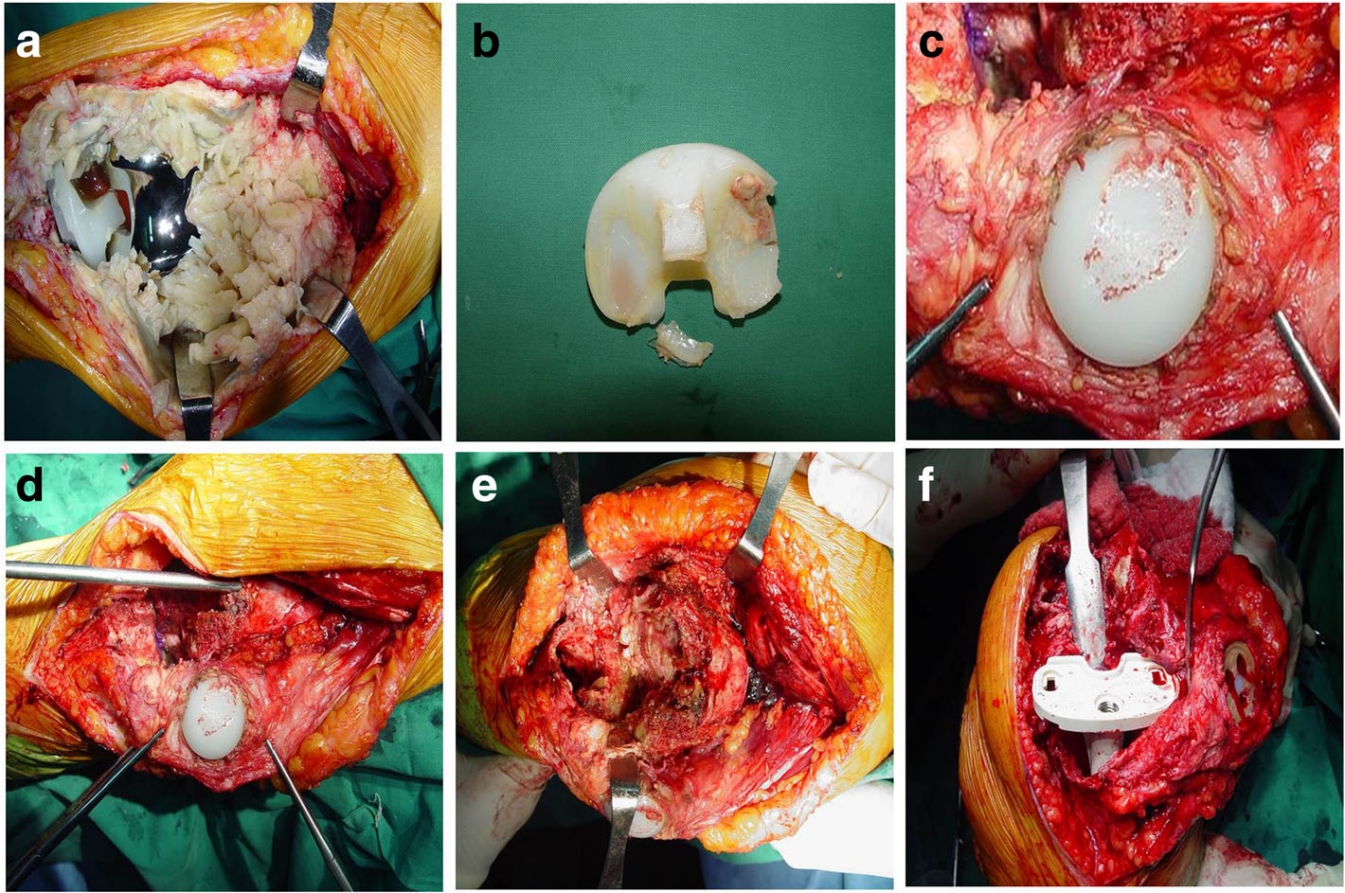
Intraoperative photographs. **a** Hypertrophic villous synovium, **b** extensive wear, delamination and deformation of the polyethylene insert distributed asymmetrically over the medial and lateral sides, **c** patellar polyethylene wear, **d** extensive bone loss in the anterior distal femur, **e** uncontained bone defect of the entire distal femoral condyle, **f** uncontained bone defect of the proximal tibia

In order to prevent a skin problem, an anterior midline incision was performed over the previous incision. A medial parapatellar arthrotomy was performed, and hypertrophic synovium was excised from the suprapatellar recess and internal and lateral gutters. The hypertrophic synovium was removed thoroughly to prevent later inflammation or dissociation. Then, the patella was everted, and the knee was flexed to 90°. During surgery, soft tissues and attached bones were preserved except hypertrophic synovium. Operative findings showed hypertrophic villous synovium, loosening of prosthesis (Fig. 2a), and wearing of tibial (Fig. 2b) and patellar polyethylene (Fig. 2c). Debridement revealed about 10 cm of extensive, anterior distal femoral cortical bone loss (Fig. 2d) and an uncontained type III defect of the entire femoral condyle (Fig. 2e). In addition, the proximal tibia had a massive, uncontained type IIA defect, according to the Anderson Orthopaedic Research Institute classification (Fig. 2f).

**Fig. 3:**
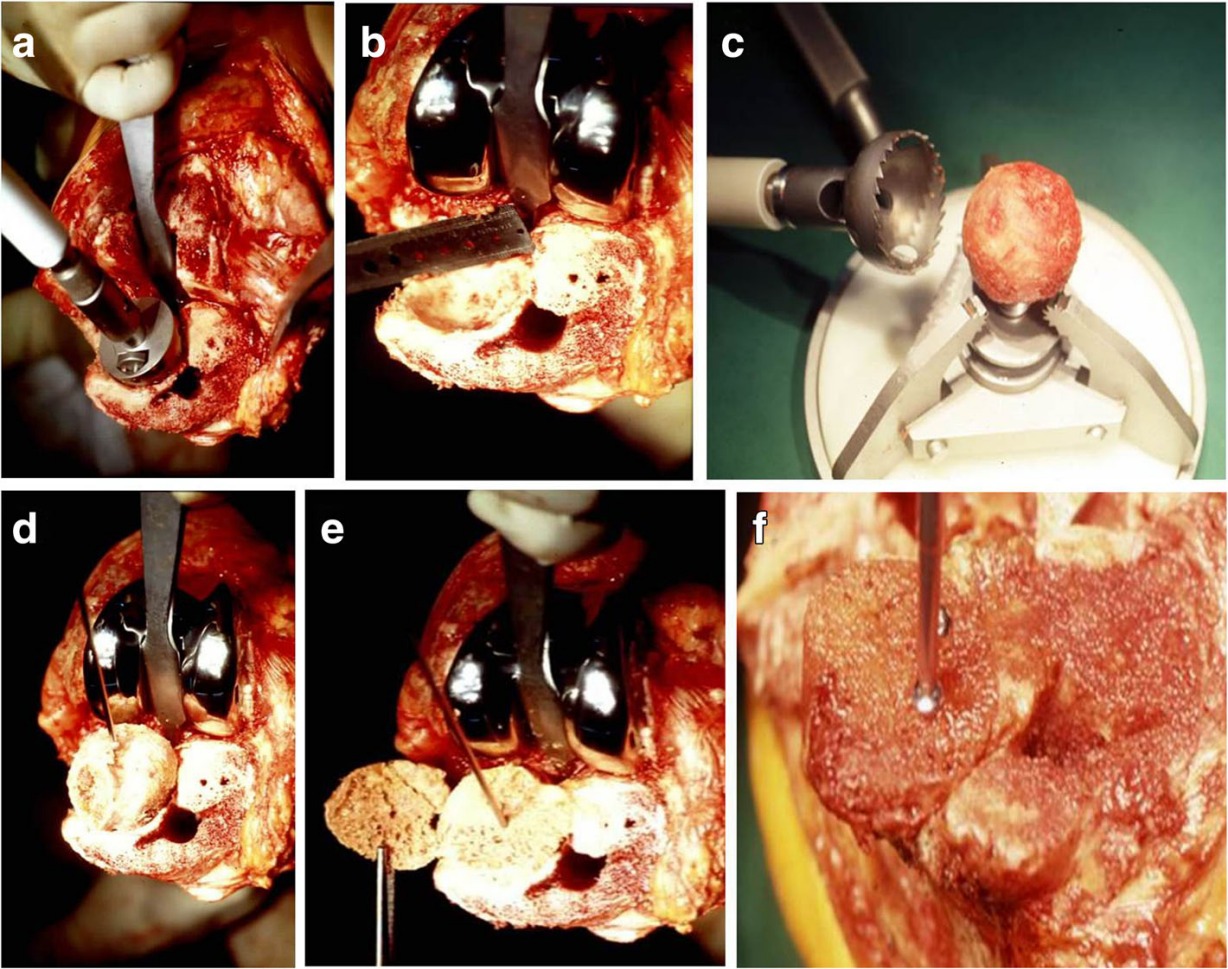
Surgical procedures on the tibial side. **a** Removal of sclerotic areas using acetabular reamer, **b** checking the defect size, **c** denuding femoral head cartilage using milling system, **d** impaction of femoral head allograft, **e** allograft resection along the tibial surface, **f** allogenic bone grafting and fixation with screws

A fresh-frozen femoral head allograft was used to fill in the proximal tibial bone loss and restore the tibial joint line; 10 mm medial and lateral proximal tibial metal blocks were used for reinforcement. In order to implant the femoral head allograft, cortical bone and cartilage were removed until cancellous bone was exposed (Fig. 3a). Next, the shape and size of the graft and bone defect sites were designed (Fig. 3b). The surface of the femoral head allograft was removed using a bone mill (Fig. 3c). The reaming exposed a trabecular structure that rapidly unites with host bone by ingrowth of woven bone, and provided an ideal surface for interdigitation of cement between the graft and implant. Bone lost in the proximal tibia and sclerotic areas was trimmed to an appropriate size, taking care not to damage cortical bone with the acetabular reamer. Allogeneic bone was designed to be 1 to 2 mm larger than the implant, allowing for impaction during implantation (Fig. 3d). The allograft was resected along the tibial surface (Fig. 3e) and internal fixation was performed using a screw (Fig. 3f); the screw was completely inserted vertically under the prosthesis to avoid contact with the prosthesis.

**Fig. 4:**
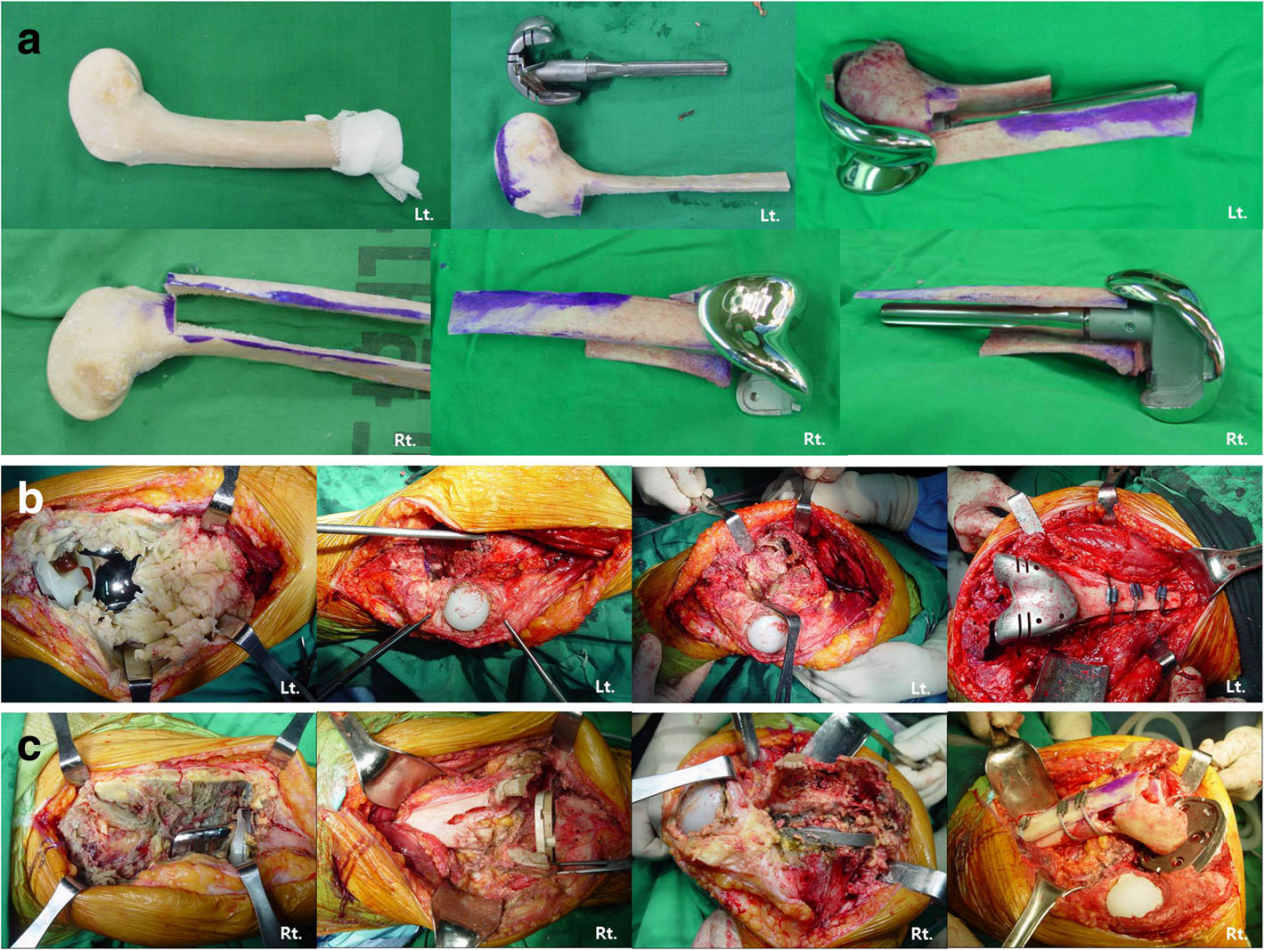
Surgical procedures on the femoral side. **a** Step-cut prepared distal femoral allografts and prepared composite distal femoral allograft with LCCK implant, using APC (allograft–prosthesis composite). **b** After extensive removal of the hypertrophic synovium of the left knee, extensive bone loss of the distal femur was observed and APC was fixed to the host bone with a Dall-Miles cable. **c** The right knee was similar operative finding to that of the left knee and APC was fixed to the host bone in the same way as the left knee

**Fig. 5:**
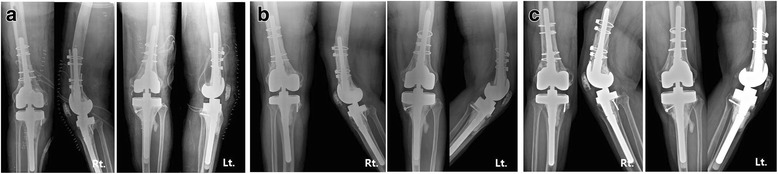
A series of radiographs. **a** anteroposterior and lateral radiographys of both knees at post RTKA, **b** 5 years follow-up, **c** 10 years follow-up showing good allograft incorporation

At another table, allografts were resected to match the prosthesis, and the grafted portions were designed in a step-cut to structurally stabilize the graft (Fig. 4a). Similarly, the host bone was designed to be step-cut to engage the allografts. Implants were inserted and allografts were attached to the host bone to confirm flexion and extension intervals, rotational alignment, and overall basic alignment. Soft tissue balance was performed to make the flexion and extension gap equal. After the trial, the cement is used to locate the prosthesis in structural allogeneic bone. Cement was used only between the constrained condylar knee stem and structural allogeneic bone, not between structural allogeneic bone and host bone. Once the cement had set, the construct was implanted with the full assembly, matching the two step-cuts. The residual host femur, with its ligaments and other soft tissues attached, was wrapped around the allograft host junction to serve as a living bone graft. Then, APC was fixed with cerclage cable (Dall-Miles®Cable System, Stryker, Mahwah, NJ, USA) (Fig. 4b, c). Using metal plates or screws can create many holes and lead to fractures. Step-cut resection and press-fit fixation were adequate to secure the allogenic bone. The extensor mechanism alignment and tracking were checked, and the wound was closed in layers. A Robert-Jones dressing was used after surgery. Post op radiographs showed no specific findings (Fig. 5a). Quadriceps strengthening and continuous passive motion exercises were started 2 days after surgery. No weight-bearing was allowed for 6 weeks, followed by 6 weeks of partial weight-bearing with crutches and a brace, then full weight-bearing with a walker starting at 12 weeks postoperatively.

After 6 weeks right revision TKA was performed using NexGen^Ⓡ^ LCCK with the same technique. Rehabilitation for the right knee was similar to that for the left knee. No signs of complications were found in follow-up examinations and post-operative radiological examinations as of February 2016 (Fig. 5b, c). The patient walked with full weight-bearing and had complete incorporation of allograft and host bone, with no signs of osteolysis. Active ROM was 0–90° in the left knee and 0–100° in the right knee. At the postoperative HSS score increased from 25/38 to 80/86. The patient is in satisfactory condition and had a in normal daily life.

## Discussion and conclusions

In this case, our patient presented with a unique problem, having severe bone loss in the both femoral and tibial aspects of both knees. Bone loss found during RTKA is caused by several causes, including; malalignment, insufficient soft tissue balance, improper cement use, asymmetric load due to improper prosthesis design, and foreign body reaction (from prosthesis wear particles); the resulting osteolysis, stress-shielding effect, and loosening of the prosthesis can result in bone loss and infection [3]. Few options are available to the surgeon for reconstruction of massive bone defects surrounding a failed TKA.

The application of allografts in RTKA is an attractive option. The use of femoral head allografts for the management of large bone defects in RTKA has been reported [4, 5]. There are few studies that simultaneously reconstruct large bone defects of distal femur and proximal tibia using allografts in RTKA [6]. Use of APC for distal femoral and massive proximal tibial allografts proved to be a successful mode of treatment with distinct advantages. In the case of relatively small size bone defects, filling a cement, impaction bone graft or metal augment can be used. In this case, this method could not be used because it was an uncontained type bone defect of entire femoral condyle. And compared to rotational hinge prosthesis and megaprosthesis, which are commonly thought of as large bone defects in general APC offers great healing capacity in terms of attaching to the host bone, which contributes to avoid massive rotational stress between them. Also, in our case, the anterior cortex of the distal femur was too slender and a rotating hinge prosthesis was not appropriate. In the megaprosthesis has the disadvantage of additional bone resection, reconstruction of the patella tendon may be difficult when using the proximal tibial component, and it is relatively difficult to preserve the original joint line. In addition, since the host bone is designed according to the prosthesis, bone loss may be greater than APC, which designs the prosthesis according to the host bone. Therefore, we used APC and there was no problem after 10 years of follow-up. However, disadvantages are not commonly available, the early recovery of range of motion and slower full weight-bearing compared to other methods. So, this case was non weight-bearing for 6 weeks after surgery, followed by 6 weeks of partial weight-bearing, then full weight-bearing with a walker starting at 12 weeks postoperatively.

Griffin et al. [7] reported that the second-generation design had a wear-related failure rate of 1.1%, compared with 8.3% in the first-generation design. Moreover, 10-year survival was was 97% with the second-generation design, compared with 87.7% for the first-generation. Peter et al. [8] also reported that wear of polyethylene could cause osteolytic lesions around the prosthesis, and this could cause eventual failure of TKA. We also observed severe polyethylene wear, despite proper soft tissue balance, femur-tibia angle, and cement use in our case. The polyethylene used in this study is a first-generation product, and osteolysis in our study seems to be one of the causes of polyethylene wear.

The thickness of polyethylene is a key factor determining the distribution of contact stress, which is inversely proportional to the degree of early wear [9]. We thought that the patient’s relatively young age (59 years old) and active lyfestyles, as well as use of 8 mm-thick polyethylene, could have caused polyethylene the wear in our case. One study reported that contact stress in polyethylene implants increases rapidly as the thickness of the implant decreases [10]. For thin polyethylene inserts, a slight further reduction in thickness increases the contact stress significantly. The study concluded that a polyethylene thickness of more than 8 mm should be used in TKA. Polyethylene inserts less than 10 mm in thickness were associated with early fatigue failure. Therefore, a thickness of at least 8 mm, but preferably 10 mm, is recommended for TKA. We used 17 mm polyethylene in RTKA. Polyethylene wear and osteolysis were not observed 10 years after surgery.

**Fig. 6:**
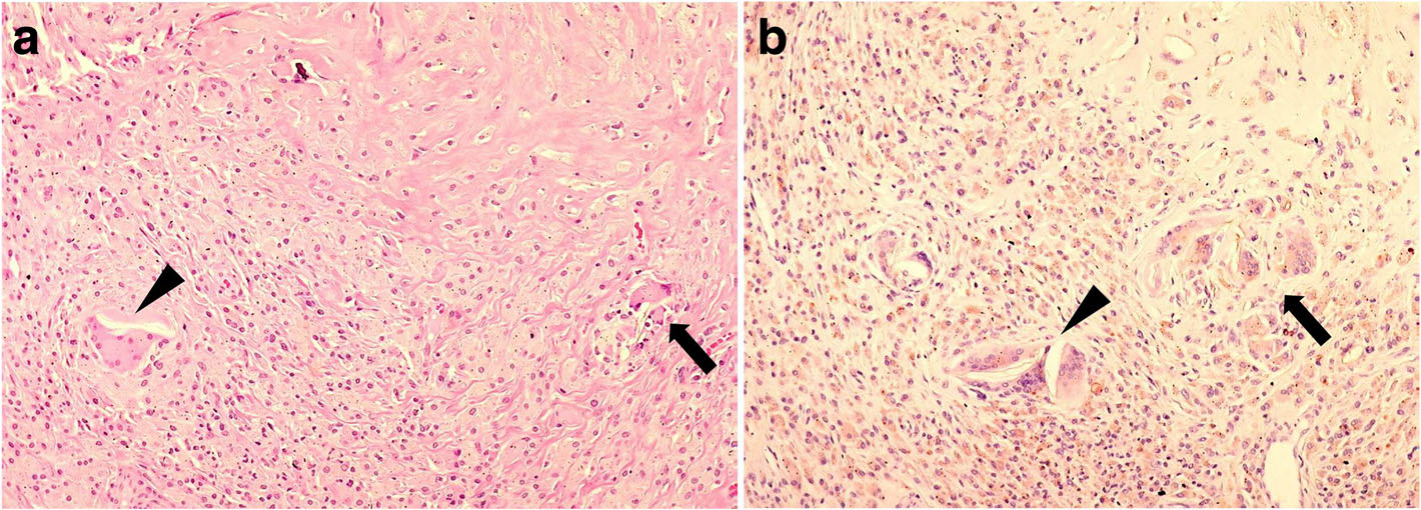
Histology of synovial tissue with macrophages, giant cells (black arrows), and giant cell- polyethylene particles (black arrowheads). **a** Hematoxylin-eosin staining (× 10), **b** Immunohistochemical staining for CD-68 (× 10)

Analyses of periprosthetic tissue retrieved during revision of failed total joint replacements showed that ultra-high-molecular-weight polyethylene (UHMWPE) wear debris is the most frequent type of debris around failed hip, knee and shoulder total joint replacements, whether or not the implants were cemented [11]. Macrophages activated by wear particle debris release an array of cytokines and proinflammatory mediators in joint fluid. This leads to recruitment, proliferation, differentiation and maturation of osteoclast precursors, and subsequent bone resorption eventually leads to implant loosening [12]. In our study, macrophages, giant cells, and foreign material, thought to be polyethylene particles, were found in inflamed synovial tissue (Fig. 6).

The use of APC enabled a stable and durable reconstruction in this uncommon presentation with large femoral bone deficiencies encountered during a RTKA. At-ten-year follow-up, we found no infection, graft failure or loosening of implants, in spite of using massive structural allografts in bilateral RTKA. Further follow-up with a larger number of patients is necessary to determine the long-term outcomes of these allografts.

## Additional files

Additional file 1: Timeline. A timeline that shows the patient’s treatment process. (DOCX 55 kb)

Additional file 2: Timeline table. A timeline table that shows the patient’s treatment process. (DOCX 14 kb)

## Abbreviations

APC: Allograft–prosthesis composites
RTKA: Revision total knee arthroplasty
TKA: Total knee arthroplasty
VVC: Varus-valgus constrained
