# Supplementary material for: A case of bilateral revision total knee arthroplasty using distal femoral allograft–prosthesis composite and femoral head allografting at the tibial site with a varus-valgus constrained prosthesis: ten-year follow up
Source: BMC Musculoskelet Disord. 2018 Mar 2;19:69. doi: 10.1186/s12891-018-1981-2 (PMC5833041; doi:10.1186/s12891-018-1981-2)
Supplement: Supplementary file 1 — Timeline. A timeline that shows the patient’s treatment process. (DOCX 55 kb) [file 12891_2018_1981_MOESM1_ESM.docx]

Timeline

A 67-year-old female with degenerative arthritis underwent both total knee arthroplasty (TKA) using the PFC modular knee system.

04/2004

Both total knee arthroplasty (TKA) using the PFC modular knee system

03/1996

P/E:
Lt. knee: Varus 10 degrees, FC 0 degree, FF 90 degrees, Internal rotation 10 degrees, HSS 38

Rt. knee: swelling, mild heat, FC 0 degree, FF 45 degrees, HSS 25

Current illness:
both knee pain since 2002.3

Diagnostic evaluation (Radiography):
Osteolysis on distal femur
Whole uncontained bone defect on medial femoral condyle & medial tibial condyle, Lt.

Diagnostic evaluation (Lab)
ESR 11, CRP 1.2

10/2005

04/2005

FU1: No change in X-ray

FU2: No change in X-ray

FU3: No change in X-ray

10/2004

07/2004

Revision TKR, Rt. with allograft

05/2004

04/2004

Revision TKR, Lt. with allograft

Diagnosis: Asceptic loosening state of TKR, both.

FU4: No change in X-ray

FU6: No change in X-ray

10/2008

FU5: No change in X-ray

10/2006

FU7: No change in X-ray

03/2009

FU8: No change in X-ray

02/2016

The patient walked with full weight-bearing and had complete incorporation of the allograft and the host bone, with no signs of osteolysis. Active ROM was 0-90 degrees in the left knee and 0-100 degrees in the right knee. The patient is in satisfactory condition and having normal daily life.
