# Supplementary material for: A case of bilateral revision total knee arthroplasty using distal femoral allograft–prosthesis composite and femoral head allografting at the tibial site with a varus-valgus constrained prosthesis: ten-year follow up
Source: BMC Musculoskelet Disord. 2018 Mar 2;19:69. doi: 10.1186/s12891-018-1981-2 (PMC5833041; doi:10.1186/s12891-018-1981-2)
Supplement: Supplementary file 2 — Timeline table. A timeline table that shows the patient’s treatment process. (DOCX 14 kb) [file 12891_2018_1981_MOESM2_ESM.docx]

| Dates | Relevant Past History and Interventions | | |
| --- | --- | --- | --- |
| 03/1996 | A 67-year-old female with degenerative arthritis underwent both total knee arthroplasty (TKA) using the PFC modular knee system | | |
| Dates | Summaries from initial and follow-up visits | Diagnostic testing | Interventions |
| 04/2004 | Current illness: both knee pain since 2002.3  Diagnosis: Asceptic loosening state of TKR, both. | Diagnostic evaluation (Radiography): Osteolysis on distal femur Whole uncontained bone defect on medial femoral condyle & medial tibial condyle, Lt.  Diagnostic evaluation (Lab) ESR 11, CRP 1.2  P/E: Lt. knee: varus 10 degrees, FC 0 degree, FF 90 degrees, Internal rotation 10 degrees, HSS 38  Rt. knee: swelling, mild heat, FC 0 degree, FF 45 degrees, HSS 25 |  |
| 04/2004 |  |  | Revision TKR, Lt. with allograft |
| 05/2004 |  |  | Revision TKR, Rt. with allograft |
| 07/2004 |  | No change in X-ray |  |
| 10/2004 |  | No change in X-ray |  |
| 04/2005 |  | No change in X-ray |  |
| 10/2005 |  | No change in X-ray |  |
| 10/2006 |  | No change in X-ray |  |
| 10/2008 |  | No change in X-ray |  |
| 03/2009 |  | No change in X-ray |  |
| 02/2016 |  | No change in X-ray |  |
| Resolution | The patient walked with full weight-bearing and had complete incorporation of the allograft and the host bone, with no signs of osteolysis. Active ROM was 0-90 degrees in the left knee and 0-100 degrees in the right knee. The patient is in satisfactory condition and having normal daily life. | | |

Timeline Table
